# On the Temperature in Children

**Published:** 1844-05-18

**Authors:** 


### ON THE TEMPERATURE IN CHILDREN.

At the Academy of Sciences 26th December, Dr. H. Roger read a memoir on the temperature of children in health and during disease. Professor Bouillaud was the first who employed the thermometer, to discover the degree of the temperature of the patient during disease. Since then Mr. Donné has published some remarks on the state of the pulse and respiration during disease, compared with the temperature. Professor Piorry in his treatise on diagnostics states, that in health it is 104° F.; in prurigo without fever 108° 75, and in typhus 117°; and lastly, in his lectures, Professor Andral pointed out the state of the temperature in adults. The end the author proposes to attain is, 1st. to point out the modifications produced by disease in the temperature of children. 2nd. to search out the laws which may be deduced from these facts; and 3rd. To make use of the laws for the diagnostic. But before indicating the state of the temperature of the child during disease, it is necessary to fix precisely the degree when in health, thus, *on coming into the world*, the thermometer placed in the axilla marked in one case 100° and in another 98°. *Three minutes after* it descended to 96° 80, then 95° 90, and finally 95° 45. The next day it was as on coming into the world. On thirty-three children from one to seven days old, the average was 98° 60; a greater activity in the circulation, male sex, &c. seemed to produce a slight elevation. On twenty-five, from four months to fourteen years old, the maximum was 100°, the minimum 98°. As to the circulation, digestion, and respiration, they seem to be without any influence.—*London Med. Times.*

